# A linearly decreasing deterministic annealing algorithm for the multi-vehicle dial-a-ride problem

**DOI:** 10.1371/journal.pone.0292683

**Published:** 2024-02-08

**Authors:** Amir Mortazavi, Milad Ghasri, Tapabrata Ray

**Affiliations:** School of Engineering and Technology, UNSW Canberra, Canberra, ACT, Australia; Cyprus International University Faculty of Engineering: Uluslararasi Kibris Universitesi Muhendislik Fakultesi, TURKEY

## Abstract

Dial a ride problem (DARP) is a complex version of the pick-up and delivery problem with many practical applications in the field of transportation. This study proposes an enhanced deterministic annealing algorithm for the solution of large-scale multi-vehicle DARPs. The proposed method always explores the feasible search space; therefore, a feasible solution is guaranteed at any point of termination. This method utilises advanced local search operators to accelerate the search for optimal solutions and it relies on a linearly decreasing deterministic annealing schedule to limit poor jumps during the course of search. This study puts forward a systematic series of experiments to compare the performance of solution methods from various angles. The proposed method is compared with the most efficient methods reported in the literature i.e., the Adaptive Large Neighbourhood Search (ALNS), Evolutionary Local Search (ELS), and Deterministic Annealing (DA) using standard benchmarks. The results suggest that the proposed algorithm is on average faster than the state-of-the-art algorithms in reaching competitive objective values across the range of benchmarks.

## Introduction

Dial-a-Ride Problem (DARP) finds various applications in the transport domain, including door-to-door transit services for the elderly and disabled [1], airport transfer services for passengers with restricted mobility [2], inter or intra-hospital transport services for patients, companions, and healthcare staff [3–8], functioning as public transport or feeder lines in low-demand times or areas [9–11]. DARP can be viewed as a generalisation of the classic pickup-and-delivery problem with time windows (PDPTW) [12, 13], and in 2003, Cordeau and Laporte [14] presented a comprehensive definition known as standard DARP. The standard DARP involves fulfilling a set of transport requests, where each request has specified origin and destination locations, along with a desired arrival or departure time. Passengers are transported by a limited number of vehicles with the objective of minimizing certain criteria while adhering to a set of constraints related to passengers’ convenience. Since then, several extensions have been proposed for the standard DARP, including considering passengers or vehicles with heterogeneity [6, 15, 16], multiple depots [17, 18], or stochasticity [19, 20] and real-time and dynamic scheduling [21]. These extensions provide a more realistic model for practical applications, but solving large-scale DARP remains a challenging task due to the exponential increase in computational time.

The solution methods for DARP can be broadly categorised into exact and meta-heuristic methods. Exact methods have the capability to identify global optima, but they are often time-consuming and impractical for large-scale DARPs. On the other hand, meta-heuristic methods cannot guarantee finding the global optimum, but they can efficiently identify high-quality solutions within a relatively short period of time [17, 22, 23]. Due to Np-hardness of DARPs [12, 23] and the high computational time requirements of exact methods for solving large scale problems [24], a significant portion of optimising research efforts has focused on proposing efficient and fast heuristic and meta-heuristic algorithms.

various algorithms have been developed for solving DARP, including individual-based algorithms, such as Tabu search [14], simulated annealing [2], neighbourhood search [25–29], and population-based algorithms such as genetic algorithm [30–34], and the bee algorithm [35]. Among these algorithms, several have proven to be more efficient compared to other algorithms in solving DARP instances, such as the deterministic annealing algorithm by Braekers, Caris [17], the adaptive large neighbourhood search by Gschwind and Drexl [29], and the evolutionary local search by Chassaing, Duhamel [36]. These methods are able to solve instances with 144 passengers and 13 vehicles within a few of minutes. However, real-world scenarios often involve significantly larger numbers of passengers and more complex networks. For instance, in a small city like Canberra, Australia, with approximately 0.5 million people, the public transport system comprises nearly 60 bus routes and a light rail line, serving around 30 thousand passengers daily [37]. Additionally, the list of requests in such scenarios is not fixed and may be affected by disruptions in the transportation network. Therefore, replacing even one feeder line of the public transport system with a DARP service requires further advancements and improvements in the solution methods to handle the increased complexity and scale of the problem.

The main contributions of this study are two-fold. Firstly, the proposed Linearly Decreasing-Deterministic Annealing (LD-DA) algorithm adopts a novel threshold adjusting approach, resulting in high-quality solutions for DARP in significantly shorter computational time. Secondly, the LD-DA algorithm can generate feasible solutions quickly right after initialisation, making it adaptable for real-time problems. These achievements are enabled by five key components that enhance the algorithm’s efficiency and effectiveness.

- First, we adopt the initialisation method proposed by Mortazavi, Ghasri [38] to generate feasible initial solutions. This initialisation method uses a greedy approach to construct vehicle tours to serve the requests without violating the constraints. Using this method, the optimisation phase always starts with a feasible solution, and it only searches the feasible space. Therefore, the optimisation phase of the algorithm does not require any penalisation for infeasibility. Besides, a feasible solution is guaranteed at any point the algorithm is terminated after initialisation.
- Second, we utilise advanced local search operators to generate new solutions in the optimisation phase, which increases the rate of convergence. The local search operators in the previous studies are heavily focused on identifying the best insertion for randomly selected requests [e.g., 17, 32], whereas in this study, the requests are selected based on the cost they impose on the existing solution.
- Third, we propose a stepwise evaluation approach to evaluate feasibility of newly generated solutions. Checking feasibility of solutions can be a time-consuming process, due to the time-window and ride-time constraints of DARP [23]. In this study, all developed local search operators use the stepwise solution evaluation mechanism, to reduce number of time constraint checks.
- Fourth, a new threshold mechanism, called Linearly Decreasing threshold adjusting (LD-threshold adjusting), is proposed. Unlike the existing threshold adjusting methods, in this threshold adjusting mechanism the maximum allowable value for the threshold gradually decreases to a pre-specified value. To accept non-improved generated solutions, existing DA algorithms use a threshold accepting mechanism, the value of which converges to zero and is reset to a randomly selected number between zero and its initial value several times during the optimisation process [39]. In this study, we adjust the maximum allowable value during the optimisation process. While this mechanism allows the solution method to escape local optima, it constrains the jump size as it converges.
- Fifth, to evaluate the efficiency of methods and determine their ability in solving DARPs, a systematic comparison is necessary. Previous studies compare the solution methods’ performances using reported objective functions and computational times. However, it is crucial to ensure the exact same implementation is used to have a fair comparison. Besides, other important aspects such as convergence rate, the number of failures in obtaining pre-specified targets, and potential impacts of stochasticity should also be considered in the evaluation process as well. This paper presents a more comprehensive comparison approach (discussed in Section ‎5), which leads to a more accurate and insightful comparison across the solution methods.

The proposed method shows a comparable performance in obtaining competitive solution quality, while it stands out by significantly reducing computational times. Additionally, the proposed method manages to introduce new-best solutions for some instances.

The rest of this article is organised as follows. Section ‎2 provides a review of previous studies in the context of DARP. The problem description is presented in Section ‎3. Section ‎4 provides the details on the structure and implementation of the proposed meta-heuristic DA, and Section ‎5 reports the performance of the proposed DA on the benchmarks. Finally, Section ‎6 summarises the findings of this study and provides suggestions for future research.

## Background

DARP is an application oriented mathematical problem with ample applications in a large variety of contexts [22, 23]. The standard DARP, defined comprehensively by Cordeau and Laporte [14], has been extensively studied [12, 17, 25, 35, 40–43]. The standard DARP involves serving a set of requests with a fleet of limited size, where requests have origin and destination locations, desired arrival or departure times, and limits on maximum ride time and route duration. A given number of vehicles with limited capacity are used to serve the requests, and the objective is to minimize the total travel cost required to serve all requests.

One research direction in the DARP literature is the expansion of the standard DARP to include practical elements. Early studies by Xiang, Chu [44] and Wong and Bell [16] added heterogeneity of passengers and vehicles to the standard DARP. This extension has been widely used in subsequent research [12, 17, 18, 28, 35]. Other studies have explored different options for dispatching or ending vehicle tours, such as single depot, multi depot, and free depot [12, 17, 18, 35]. Schilde, Doerner [19], Schilde, Doerner [20], and Zhao, Poon [8] incorporate network disruptions and time-dependent travel costs, which improved the ability of solution methods to deal with stochastic and dynamic scenarios based on historical information. Azadeh, Atasoy [45] extended standard DARP by integrating passengers’ behaviour in the operational planning using choice models, where passengers are able to select between shared and private vehicles.

One area of research within the domain of DARP is focused on developing effective methods to solve the problem. Finding high-quality solutions in a short amount of time has been a challenge, especially for real-time applications with limited response time [23]. Exact and heuristic/meta-heuristic methods are two general categories of proposed solution methods for solving DARPs. Several researchers have proposed exact methods to solve DARPs, such as Cordeau [40] developed a three-index branch-and-cut formulation to solve instances with 48 requests and 4 vehicles with CPU times of around 4 hours. Ropke, Cordeau [43] introduced a two-index branch-and-cut formulation which was able to solve instances with up to 96 requests and 8 vehicles in about 2 hours. Braekers, Caris [17] proposed their branch-and-cut method based on the two-index formulation. They adopted binary variables for solving the standard DARP and binary and continuous variables for heterogeneous DARPs. Their algorithm could find optimal solutions for instances with 50 requests in a few seconds. However, the computational time increased dramatically for larger instances. For example the reported run time for an instance with 96 requests is almost 4 hours [17]. Although exact methods can obtain global optima, they are not practical for solving large-scale problems due to their high computational time requirements [23]. Therefore, exact methods have been adopted for solving small scale problems or validating efficiency of heuristic methods. For example, Guo, Guan [46] developed three methods, one exact and two were heuristic methods, to apply them on a real scenario in Beijing. However, this study found the proposed branch-and-cut (exact) method applicable on small-scale problem and used for validating efficiency of two other methods. In another example, branch-and-price algorithm was applied by Dou, Meng [47] for investigating the effect of demand change on the system. Due to the complexity of problem, this algorithm was only used to deal with 74 passengers benchmark problem while heuristic methods were utilised for large-scale instances.

Due to the complexity and NP-hard nature of optimising problems, meta-heuristic methods are frequently employed to address NP-hard optimisation problems, including DARPs [7, 22, 23, 48]. The primary objective of these proposed methods is to achieve high-quality solutions based on the problem’s objective function. In the literature of DARPs, various optimisation algorithms have been developed, including individual-based [2, 5, 18, 20, 25, 27, 42, 49–51], population-based algorithms [30, 34, 52, 53], and hybrid forms of algorithms [32, 35, 54–57]. The significant ones are briefly explained below. One of the early meta-heuristic methods proposed is the Tabu search introduced by Cordeau and Laporte [14]. This approach applies a single relocate neighbourhood operator along with an intra-route operator in an iterative process to generate new solutions from previous ones. Parragh and Schmid [33] proposed the hybrid column generation and large neighbourhood search algorithm, which showed slightly better solutions in significantly lower CPU times when compared to the Tabu search by Cordeau and Laporte [14]. The improvement was approximately 1 percent in the objective function value and about 50 percent in runtime. Braekers, Caris [17] proposed a deterministic annealing algorithm that considerably reduced the runtime for both homogeneous and heterogeneous DARPs. This method could achieve the same quality solutions as the previous approach for instances with up to 144 passengers and 13 vehicles in just 3 minutes, nearly 10 times faster. The proposed meta-heuristic bee algorithm and its hybrid with DA [35] or the hybrid genetic algorithm [32] slightly improved the solution quality, but their computational times were 20 to 25 percent higher than DA. Malheiros, Ramalho [12] introduced the multi-star hybrid approach, which outperformed DA (Braekers, Caris [17]) in both solution quality and computational time for instances with less than 100 requests. However, for instances with a larger number of requests, the algorithm required almost 60 percent higher computational cost compared to DA. The adaptive large neighbourhood search proposed by Gschwind and Drexl [29] benefited from a constant feasibility check procedure, resulting in slightly better objective values in almost half the computational time compared to DA. For a comprehensive review on DARP formulations and solution methods, the readers are referred to Parragh, Doerner [22], Molenbruch, Braekers [48], and Ho, Szeto [23].

The above review highlights the challenge of efficiently solving large scale DARP within a limited computational budget. The research outlined in this paper aims to address the important gap of delivering high-quality solutions for large-scale standard DARPs. The proposed algorithm not only achieves this goal but also features fast computation, making it suitable for practical applications with time restrictions. The proposed method improves upon existing DARP solutions by adjusting the local search operators and the accepting threshold mechanism in DA. In addition, the method uses a unique initialization technique to construct feasible solutions and local search operators that only search through feasible space. As a result, the algorithm can return a feasible solution at any point of termination, making it an ideal choice for time-restricted operational applications. To further evaluate the strength of the proposed method, a more systematic and intensive series of experiments are conducted. This provides more detailed and insightful assessments of the algorithm’s capabilities. By analysing the results of these experiments, we can gain valuable insights into the strengths and limitations of the proposed approach.

### Problem description

The Definition of the DARP is grounded in a transport network represented by a graph denoted as *G* = (*N*, *A*). In this graph, *N* represents the set of all vertices (N=P∪D∪{0,2n+1}) and *A* denotes the set of all arcs. *P* and *D* correspond to subsets of pick-up nodes {1.…,*n*} and drop-off nodes {*n*+1.….2*n*} nodes, respectively. Each route must originate and conclude at the depot (nodes 0 and 2*n*+1, respectively). The number of passenger requests is indicated by *n*. For each request, its origin is denoted by *i*, while its corresponding destination is denoted by *n*+*i*. Each vertex is associated with a load, denoted by *q*_*i*_, where *q*_*i*_ equals zero for the depot (*q*_0_ = *q*_2*n*+1_ = 0), equals 1 for the origins (0<*i*≤*n*), and equals -1 for destinations (*n*<*i*≤2*n*). The transportation of these requests is undertaken by a limited number of homogenous vehicles represented by *V*, where every vehicle *v* has a limited capacity of *Q*_*v*_. Additionally, every vertex is associated with a time window representing requests’ pick-up or drop-off constraints that are specified by the earliest visiting time (*e*_*i*_) and the latest visiting time (*l*_*i*_). Travelling on the arc from node *i* to node *j* comes at the travel time (travel cost) of *C*_*ij*_. At each vertex, loading or unloading operations last for a given service time *d*_*i*_. Moreover, the passengers’ on-board time of each passenger must be lower than the maximum allowable ride time (L) and vehicles’ tour duration must be below the maximum allowable tour duration (D). The following mathematical formulation of DARP is presented in Eqs (1 to 13). This formulation is based on the mixed integer formulation proposed by Cordeau and Laporte [14].


$$Min\;f(s)=\sum_{v\in V}\sum_{i\in N}\sum_{j\in N}C_{ij}X_{i,j}^{v}$$ (1)

Subject to:

$$\sum_{v\in V}\sum_{j\in N}X_{i,j}^{v}=1\;\forall i\in P$$ (2)

$$\sum_{j\in N}X_{i,j}^{v}-\sum_{j\in N}X_{i+n,j}^{v}=0\;\forall i\in P,v\in V$$ (3)

$$\sum_{j\in N}X_{0,j}^{v}=\sum_{i\in N}X_{i,(2n+1)}^{v}=1\;\forall v\in V$$ (4)

$$\sum_{j\in N}X_{j,i}^{v}-\sum_{j\in N}X_{i,j}^{v}=0\;\forall i\in P\cup D,v\in V$$ (5)

$$A_{j}^{v}\geq (A_{i}^{v}+d_{i}+C_{i,j})-M_{i,j}^{v}(1-X_{i,j}^{v})\;\forall i,j\in N,v\in V$$ (6)

$$A_{i+n}^{v}\geq A_{i}^{v}\;\forall i\in P,v\in V$$ (7)

$$e_{i}\leq A_{i}^{v}\leq l_{i}\;\forall i\in N$$ (8)

$$C_{i,j}\leq A_{i+n}^{v}-{(A}_{i}^{v}+d_{i})\leq L\;\forall i\in P,v\in V$$ (9)

$$A_{2n+1}^{v}-A_{0}^{v}\leq D\;\forall v\in V$$ (10)

$$Q_{j}^{v}\geq (Q_{i}^{v}+q_{j})-W_{i,j}^{v}(1-X_{i,j}^{v})\;\forall i,j\in N,v\in V$$ (11)

$$max\{0,q_{i}\}\leq Q_{i}^{v}\leq Q_{v}\;\forall i\in N,v\in V$$ (12)

$$X_{i,j}^{v}\in \{0,1\}\;\forall i,j\in N,v\in V$$ (13)

The objective of this problem is minimising total travel cost discuss by Eq (1). While Eq (2) checks visiting all origins and destinations of passengers, Eq (3) ensures each passengers’ origin and destination are visited by the same vehicle. Eq (4) ensures that each vehicle’s tour starts from and ends at the depot. Eq (5) conserve the flow of route. Eq (6) calculates the arrival times at any node, while Eq (7) checks destinations are only visited after their corresponding origins are visited, and Eq (8) checks the time window constraint of each node. In these equations, $A_{i}^{v}$ is the arrival time of vehicle *v* at node *i*, and $M_{i,j}^{v}\geq max(0,l_{i}+C_{i,j}-e_{i})$. Eqs (9 and 10) check the ride time constraint for each passenger and the tour duration constraint for each vehicle, respectively. Eq (11) calculates number of onboard passengers for vehicle *v* at node *j*, which is visited after node *i*. In this equation, $Q_{i}^{v}$ is the number of on-board passengers at node *i*, and $W_{i,j}^{v}$ is used for linearising equation and the validity of constraint is ensured by setting $W_{i,j}^{v}\geq min(Q_{v},Q_{v}+q_{i})$. Eq (12) ensures that the vehicle capacity constraint is respected. Finally, Eq (13) indicates that $X_{i,j}^{v}$ is a binary varibale, where it is equal to 1 if vehicle *v* travels from node *i* to node *j*, and zero otherwise.

### Methodology

Algorithm 1 presents the structure of the proposed approach which is referred as Linearly Decreasing-Deterministic Annealing (LD-DA) algorithm. LD-DA is modified version of deterministic annealing algorithm, known also as threshold accepting, is a deterministic variant of Simulated Annealing [39]. Overall, the proposed LD-DA is a local search-based iterative metaheuristic algorithm that begins with an initial solution and generates new solutions in each iteration by applying variation operators to the current solution. The new generated solution replaces the current solution if its objective value is equal to or better than the current solution’s objective value, or if the new objective value is not greater than the current objective value plus a threshold.

Algorithm 1. Implemented Meta-heuristic Deterministic Annealing with the New Developed Threshold Adjusting Mechanism

# Initialisation part: Generating an initial solution and set parameters

*x*_*initial*_: constructed solution by “Adaptive reinforced logit models”.

Set initial value of parameters:

${Adjusting}_{Iteration}=0$

$T_{max}=Coef_{T,max}\times averagearcs^{\prime }cost,T_{min}=Coef_{T,min}\times T_{max},T^{\prime }=T_{max}-T_{min},T=T_{max}$

$x_{best}=x_{current}=x_{initial}$

$for\;iteration=1\to n_{iter}:$

# Part 1: Applying local optimising operators

${Adjusting}_{Iteration}\leftarrow {Adjusting}_{Iteration}+1$

$for\;Operator_{i}\;from\{list\;ofInterRoute_{operators}\}$

$x_{new}\leftarrow apply\;Operator_{i}\;on\;x_{current}\;to\;generate\;a\;new\;solution$

$if\;x_{new}\;is\;feasible\;and\;f(x_{new})\leq f(x_{current})+T:$

$x_{new}\leftarrow apply\;local\;intra\;route\;on\;x_{new}$

*x*_*current*_←*x*_*new*_

$if\;{f(x_{current})<f(x}_{best}):$

*x*_*best*_←*x*_*current*_

***Adjusting***_***Iteration***_←0

 # Part 2: Adjusting threshold

***if Adjusting***_***Iteration***_>0:

Threshold←Threshold−(TmaxTreduction)

***if*** *Threshold*<0:

${Threshold}^{\prime }\leftarrow (1-\frac{iteration}{n_{iter}})\times (T_{max}-T_{min})$

$Threshold\leftarrow T_{max}\leftarrow (T^{\prime }+T_{min})$

$if\;{Adjusting}_{Iteration}>n_{restart}:$

*x*_*current*_←*x*_*best*_

${Adjusting}_{Iteration}\leftarrow 0$

***Return*** *x_best_*

LD-DA comprises an initialisation phase and an optimisation phase (Part 1 and 2). In the initialisation phase, a feasible solution is constructed by the adaptive reinforced logit models introduced in [38]. The initial values of the algorithm parameters are also set in this phase. The parameters of this method comprise maximum threshold (*T*_*max*_), and minimum threshold (*T*_*min*_), which are defined based on the size and scale of the instance. In this study *T*_*max*_ is defined as *Coef*_*t*,*max*_×*average arcs′cost* and *T*_*min*_ is defined as *Coef*_*t*,*min*_×*T*_*max*_. *Coef*_*t*,*max*_ and *Coef*_*t*,*min*_ where the parameters are discussed in detail in Section ‎5.2.1.

The optimisation process in LD-DA consists of two parts i.e., solution generation and threshold adjustment. In Part 1, local search operators are applied to the current solution (*x*_*current*_) to generate new solutions (*x*_*new*_). *f*(*x*) denotes the objective value of solution *x*. New solution will replace the current solution if *f*(*x*_*new*_)≤*f*(*x*_*current*_)+*T*. Since non-improved solutions can be accepted and replace current solution, the best solution (*x*_*best*_) may differ from the current solution. Therefore, once the current solution is updated, the best solution is also checked to see if updating is required *f*(*x*_*current*_)<*f*(*x*_*best*_).

Previous DARP solution methods allow for violations of one or more constraints (e.g., time window, ride time, and tour duration) in the initialisation phase or during the exploration phase. To remove infeasibility, they either implement repair operators [17, 32, 35], or penalise the objective function for infeasibility [6, 14, 25]. In this study, the initialisation phase provides a feasible solution, and in the optimisation phase only feasible solutions are accepted. Therefore, the repair operators or objective function penalisation are not required.

In Part 2, the threshold of accepting non-improved solutions is adjusted. The threshold (denoted by *T*) is used in the optimisation phase to decide whether the current solution should be replaced by a new solution. In the iterations that the best solution remains unchanged, *T* is reduced by *T*_*max*_/*T*_*reducation*_, where *T*_*max*_ and *T*_*reducation*_ are user defined parameters (Section ‎5.2.1). Once *T* becomes negative, it is reset to a positive value. In the previous applications of DA for DARP, T is reset to a random value between 0 and *T*_*max*_ [17, 58, 59]. In this study, a new mechanism is proposed, where *T* is reset to *T*_*max*_, and *T*_*max*_ linearly decreases with time. This mechanism constrains the jump size as the algorithm converges to the optimal solution. To enable the algorithm to escape from local optima, we set a minimum value for *T*_*max*_ (denoted by *T*_*min*_ in the Algorithm 1).

Since *x*_*current*_ is different from *x*_*best*_, restart mechanism is embedded in Part 2 to reset *x*_*current*_ to *x*_*best*_, if there is no improvement in the best solution for a while. Every time *T* becomes negative, number of iterations that *x*_*best*_ has remained unchanged is checked, and if it is more than considered *n*_*restart*_ number of iterations, *x*_*current*_ is replaced with *x*_*best*_. The restart mechanism gives the algorithm an ability for better exploration.

### Initialisation method

The adaptive reinforced logit model proposed by Mortazavi, Ghasri [38] is used to construct an initial solution. For single vehicle DARP, the models create a string of nodes to visit by choosing the next node with the lowest likelihood of generating an infeasible solution at each step. The probability of infeasibility is calculated using three logit models. For multi-vehicle DARP, passengers are assigned to vehicles in an iterative process. In each iteration, a passenger is randomly added to a vehicle and the logit models are utilised to construct a feasible solution for the vehicle with all passengers allocated to it so far. If a feasible route cannot be generated for the vehicle, the passenger is assigned to the next available vehicle. If there is no other available vehicle to check, the passenger will be added to a list of unassigned passengers. Once all passengers have been assigned, a heuristic insertion technique is applied to add unassigned passengers to the constructed routes at their optimal positions if the unassigned passengers list is not empty. Mortazavi, Ghasri [38] showed this initialisation method is on average 2.8 faster in constructing an initial solution compared to the Parallel Insertion Heuristic method proposed by Braekers, Caris [17].

To increase the likelihood of feasible solution construction, it is recommended to calibrate the parameters of the logit models for the specific problem. The process starts by randomly selecting 5 passengers from the smallest instance (with low number of requests) to create an initial training dataset. All possible combinations of these passengers are used to calibrate the parameters of the initial logit models. The performance of the logit models in generating feasible solutions is then evaluated by gradually increasing the number of passengers. If the success ratio drops below 25 percent, augmentation is triggered. This involves using a new training dataset that includes feasible and infeasible solutions generated by the latest trained logit models to recalibrate the parameters of the logit models. The number of passengers assigned to each vehicle is determined by the ratio of passengers to total available vehicles in the instance.

### Optimising operators

This study employs local search operators to create new solutions, including four inter-route operators (swapping, relocation, 2-opt*, and successive requests relocation) and one intra-route operator (r-5-opt).

The first operator is the **swapping** operator, which is a local search operator that randomly selects two vehicles and one request from each vehicle. The selected requests are then removed from their current vehicles and inserted into the other vehicle in their best possible positions. The requests can be selected either randomly or based on their imposed cost on the current route (with a chance of 50–50 percent). If the second option is taken, the imposed cost is calculated, and the probability of selecting requests based on their imposed costs is calculated using the roulette wheel mechanism proposed by [60].


$$Imposed\;cost\;from\;selection\;request\;i=\frac{C_{i-1,i}+C_{i,i+1}+C_{i+n-1,i+n}+C_{i+n,i+n+1}}{total\;travel\;cost\;of\;selected\;vehicle}$$ (14)

Inserting request in their best possible position uses an embedded heuristic method, which finds the feasible position for the selected request with the minimum objective function value. In this heuristic method, all possible positions need to be enumerated and the request is inserted in the best possible position (Section ‎4.3).

The second operator is the **relocation** operator. For the relocation operator, a vehicle route is randomly selected and a random set of requests (up to half of the existing requests in the route) is removed one by one and reinserted in their best position. The requests are selected based on two selection options of random selection or imposed cost (Eq (14)).

The third operator is the 2-opt* operator. In this operator, a single point crossover permutation is applied to two vehicle routes to generate two new vehicle routes. This operator was developed by Potvin and Rousseau [61] for solving routing problems with time windows. The crossover point is selected from the arcs where the vehicle is empty (empty arcs), to make sure the origin and destination of each request are going to be served with the same vehicle. Then each route is broken from the selected arc into two parts and two new routes are constructed from recombining the resulting parts, where the first part of first route is combined with the second part of second route and vice versa. Arcs are selected randomly.

The fourth operator is the **successive requests relocation** operator. In this operator, a random vehicle route is selected. The same as previous operator, empty arcs are identified. Two successive empty arcs are randomly selected. The selected arcs are removed, which breaks the route into three separated parts, one before the first removed arc, one between two removed arcs, and one after second arc. Then one of these three parts is randomly selected, and its requests are reinserted one by one in their best possible position in other vehicles.

The last adopted operator is an intra-route one, called **r-5-opt**, which is a version of k-opt operator with *k* = 5 [17]. The r-5-opt is applied once a new solution is accepted. First, a route is randomly selected and the r-5-opt is applied to the selected route. For an existing sequence of vertices in a vehicle route, this operator examines all possible ordering of every four successive vertices in the sequence, in an attempt to reduce the cost while maintaining feasibility.

### Solution evaluation

The proposed LD-DA only explores the feasible search space. Therefore, the feasibility of new generated solutions must be checked. Checking the feasibility of solutions is time consuming, mainly because of the time constraints in DARP [17, 23]. Parragh, Doerner [25] developed an eight-step evaluation scheme for the standard DARP, which first computes the visiting time service for each vertex and compares it with time window constraints. One can encounter one of three conditions. First, if visiting time is less than the earliest visiting time, then waiting time is calculated as the difference between visiting time and earliest visiting time and the visiting time is set to the earliest visiting time. Second, if visiting time is between the earliest and latest visiting time, then waiting time is set to zero. Third, if visiting time is greater than the latest visiting time, then the solution is infeasible and is discarded. If the solution meets the time window constraints, then, the requests’ on-board time is computed in the order of the requests’ destination position in the route. If any ride time violation is found, the scheme tries to adjust the solution and fix the violation by delaying the visit for the vertices between the origin and destination. The delays are limited to the minimum difference between the latest visiting time and visiting time of all vertices between the origin and destination of the request violating the ride time constraint. If adjusting is not possible, the solution is labelled as infeasible and is discarded. For more details about the eight-step evaluation scheme refer to Parragh, Doerner [25].

As the reinsertion part of all operators in our study aims to identify the best possible position, checking the feasibility of numerous potential solutions will be necessary. However, using the eight-step evaluation scheme to evaluate feasibility could be time-inefficient due to the large number of solutions that need to be evaluated. Therefore, the following screening process is proposed. This screening process removes those solutions that cannot be adjusted by the eight-step evaluation scheme, and it sorts the solutions based on their objective values. The eight-step evaluation scheme is then applied from the top of the list and once a feasible solution is found, the rest of the solutions are discarded. The details of the screening process are as follows.

1. All potential combinations for inserting a request are constructed by inserting the origin in any position in the route, and the destination in any position after the origin.
2. Combinations are first checked based on the objective value. LD-DA accepts a new solution only if it does not deteriorate the objective function more than the threshold (Section ‎4). This step removes all combinations that will not be accepted due to their high objective value irrespective of their feasibility.
  - First, the objective value of a combination which only visits the origin is calculated. If the objective value does not pass the threshold accepting function, there is no point in checking combinations that visit the destination as their objective value will be even higher, therefore they all will be discarded.
  - The combinations with both origin and destination will be checked, and those combinations that do not meet the threshold accepting function will be removed.
3. The list of remaining combinations is sorted in an ascending order of the objective value.
4. Then, the combinations are checked based on the following criteria. The first combination that satisfies both criteria will be returned as the best solution and the process is terminated.
  - Does the combination meet the capacity constraints?
  - Does the combination pass the eight-steps evaluation scheme.

### Computational experiment

In this section, we evaluate the performance of our proposed meta-heuristic LD-DA by comparing it with three other state of the art algorithms for solving the DARP: Adaptive Large Neighbourhood Search (ALNS) by Gschwind and Drexl [29], Evolutionary Local Search (ELS) by Chassaing, Duhamel [36], and Deterministic Annealing (DA) by Braekers, Caris [17]. We begin by comparing the LD-DA with ALNS and ELS in terms of solution quality and runtime. Next, we conduct a more systematic series of experiments to compare the performance of LD-DA and DA from different angles. We also investigate the effectiveness of the linearly decreasing threshold developed in our study. The computational experiments were conducted on a laptop with a 2.3 GHz Core processor and 16 GB RAM, with LD-DA implemented in C++. The used source code of DA from Braekers, Caris [17] in the next sub-sections is made available by the authors.

### Benchmark

The benchmark developed by Cordeau and Laporte [14] is one of the most widely used and largest benchmarks for standard DARP. The benchmark includes 20 randomly generated instances (labelled as R1a-R10a and R1b-R10b) with a range of 24 to 144 passengers and 3 to 13 vehicles. Each vehicle has a capacity of 6 passengers. Tour duration and ride time limitations are equal to 480 and 90 units, respectively. Half of the requests have a constrained pick-up time window and half of them have a constrained drop-off time window. In the first ten instances (R1a-R10a), time windows are smaller (between 15 to 45 units), while in the second ten instances (R1b-R10b) time windows are larger (between 30 to 90 units).

### Meta-heuristic LD_DA performance

#### Initial parameters

In the first experiment, all algorithms are compared based on the quality of solutions. The performances of algorithms depend on a number of user-defined parameters. For the DA proposed by Braekers, Caris [17], user-defined parameters are set to recommended values in their study. For ALNS and ELS, we did not undertake any programming and we obtained the performance metrics from the corresponding papers. The proposed LD-DA algorithm contains five user-defined parameters. These parameters are the number of iterations (*n*_*iter*_), maximum threshold (*T*_*max*_), minimum threshold (*T*_*min*_), threshold reduction parameter (*T*_*reduction*_), and restart iteration parameter (*n*_*restart*_). The parameters of *T*_*max*_ and *T*_*min*_ are defined relative to the scale of problem and as functions of the average arc travel cost, where *T*_*max*_ = *Coef*_*t*,*max*_×*average arcs*′*cost* and *T*_*min*_ = *Coef*_*t*,*min*_×*T*_*max*_. All these parameters’ values (except *n*_*iter*_) are set based on the sensitivity analysis (discussed in S1 Appendix), and their values are as follows:

- *Coef*_*t*,*max*_ = 2,
- *Coef*_*t*,*min*_ = 0.2,
- *n*_*restart*_ = 300,
- *T*_*reduction*_ = 300,

In the following subsections, the value of *n*_*iter*_ is set in different ways according to the experiment, which is explained in each corresponding subsection.

#### Comparing performance of LD-DA with successful methods

In this subsection, the proposed LD-DA is compared with the ALNS and ELS, which along with the DA, are considered as the state-of-the-art algorithms for the solution of DARPs. To conduct this comparison, first, the termination of the LD-DA must be determined. To do so, various number of iterations are considered and the performance of the LD-DA for several instances on different termination condition is collected. The results show performance of LD-DA remains relatively stable after 50,000 iterations. Thus, this termination is set for comparing the performance of the LD-DA with the other methods. Detailed results on the performance of the LD-DA for other instances can be found in Table 10 in the S2 Appendix.

Table 1 provides an overview of the performances achieved by our proposed LD-DA method. It also presents a comparative analysis with the outcomes of ELS and two distinct executive versions of ALNS, utilising a dataset comprising 20 instances of the Cordeau and Laporte [14] benchmark. The table presents the best-obtained objective in the "Best" column, the average objective in the "Avg" column, and the average required computational time in the "CPU" column, measured in seconds. For fairness and consistency purposes, we executed our algorithm five times, aligning with the reported number of run for other algorithms and compare the results with reported results in their studies [29, 36].

**Table 1:** Comparing performances of LD-DA with ELS and ALNS on 20 standard DARP instance of [14].

Instance	*BKS* ^1^	ELS			ALNS						LD_DA (50 k iterations)		
					*Pure* ^2^			Best *configuration*^2^					
		Best	Avg	CPU	Best	Avg	CPU	Best	Avg	CPU	Best	Avg	CPU
pr01	190.02	190.02	190.02	15.00	190.02	190.02	8.20	190.02	190.02	14.50	**190.02**	190.02	5.79
pr02	301.34	301.34	301.34	75.00	301.34	301.34	16.70	301.34	301.34	38.40	**301.16**	302.50	13.85
pr03	532.00	532.43	533.86	138.00	532.00	532.37	28.80	532.00	532.00	55.20	**529.15**	537.62	11.29
pr04	570.25	570.54	574.47	442.20	571.41	574.34	48.10	570.29	570.86	142.20	576.81	581.87	14.52
pr05	625.64	630.82	637.59	724.20	632.44	635.26	69.80	629.14	632.67	296.22	636.61	644.43	22.72
pr06	783.78	792.80	796.10	1315.20	792.97	795.25	95.30	788.86	790.25	370.00	796.72	811.24	27.30
pr07	291.71	291.71	292.96	28.20	291.71	291.71	10.20	291.71	291.71	19.00	**291.71**	291.71	8.25
pr08	487.84	491.60	493.16	160.80	491.97	493.36	30.10	489.89	491.44	70.70	491.74	501.86	10.40
pr09	653.94	672.86	681.35	671.00	660.55	665.63	55.30	658.90	661.37	151.20	662.31	668.91	9.81
pr10	845.47	857.36	860.68	1279.80	855.73	862.35	96.50	854.60	858.65	369.10	866.44	880.42	23.57
pr11	164.46	164.46	164.46	16.80	164.46	164.46	9.40	164.46	164.46	16.50	**164.46**	164.51	4.77
pr12	295.66	295.66	295.72	82.20	295.96	296.52	18.80	295.66	296.18	44.50	**295.66**	297.99	17.03
pr13	484.83	489.00	490.70	222.20	485.82	488.68	33.80	484.83	485.15	94.90	485.47	493.57	15.81
pr14	529.33	531.08	531.98	612.00	532.16	534.85	57.00	530.88	531.94	220.00	536.12	544.92	23.87
pr15	573.56	578.44	580.23	1195.80	582.07	584.07	89.00	576.88	577.27	469.60	584.71	592.16	36.38
pr16	725.22	731.25	736.59	1939.20	739.67	742.80	117.60	737.09	740.12	687.90	738.14	749.56	40.92
pr17	248.21	248.21	248.21	34.80	248.21	248.21	11.40	248.21	248.21	22.10	**248.21**	251.98	1.61
pr18	458.73	461.21	462.40	259.20	461.12	463.51	35.00	461.48	461.64	99.70	463.52	466.63	12.87
pr19	592.23	595.39	597.53	745.80	596.91	599.33	69.60	594.14	596.02	343.50	604.52	611.34	12.04
pr20	783.81	796.60	803.99	1887.00	785.70	793.10	114.70	784.57	790.01	472.20	796.12	805.72	32.53
**Average**	506.90	511.14	513.67	592.22	510.61	512.86	50.77	509.25	510.57	199.87	512.96	519.45	17.27

1- BKS: Best Known Solutions, provided by [29].

2- Pure and Best configuration are two executive recommended versions of the ALNS proposed by Gschwind and Drexl [29], which interested readers for having more information about the differences are referred to this study.

The results reveal that LD-DA yields equal or enhanced solutions compared to the Best-Known Solutions (BKS) in 7 instances. Additionally, LD-DA demonstrates the capacity to generate solutions of comparable quality for the remaining instances, exhibiting an average gap of 0.91 percent when compared to BKS. In contrast to the ELS method proposed by Chassaing, Duhamel [36], the proposed LD_DA method effectively showcases its ability to provide high-quality solutions within an impressively short computational timeframe. The gap in best solutions between these two methods is approximately 0.27 percent, while LD_DA displays a remarkable speed improvement by being 28 times faster in achieving solutions. A more detailed comparison can be found in Table 11 in S2 Appendix. Furthermore, LD_DA’s performance is assessed against the two proposed versions of ALNS by Gschwind and Drexl [29]. In comparison to the Pure and Best configuration ALNS, LD_DA manages to secure solutions of comparable high quality with gaps of 0.34 and 0.56 percent, respectively. Notably, LD_DA outperforms these configurations in terms of computational efficiency, operating at speeds that are 3 and 10 times faster, respectively. For a comprehensive analysis of LD_DA’s comparison with the Pure and Best ALNS configurations, please refer to Tables 12 and 13 in S2 Appendix.

This comparison clearly demonstrates the effectiveness of the proposed method in outperforming the other three state-of-the-art methods in terms of the computational time required to achieve high-quality solutions. This superiority signifies that the proposed method holds a higher level of reliability when it comes to being adopted for generating high-quality solutions in larger-scale problems (like real-world scenarios) within a practical computational time.

#### Comparing proposed LD-DA with DA

Numerical experiments conducted in Section ‎5.2.2 demonstrated the competitiveness of the proposed LD-DA with the cutting-edge solution methods. However, as mentioned in the introduction, a more detailed comparison is required for determining the strength and weakness of any new solution method. To further demonstrate the superiority, a more robust and detailed comparison is necessary, which requires more insightful experiments. To achieve a fair comparison between the two solution methods, both methods must be executed on machines with identical specifications. Since the source code of DA was provided to us by the authors, Braekers, Caris [17] it was utilised for the rest of experiments in this study. In section ‎5.2.2 we run the algorithm five times to keep it consistent and comparable with how previous studies present their findings. However, as we have access to the DA method source code, we decided to increase the number of iterations to 21 in order to enhance the robustness of the findings. In the following sub-sections, the proposed method is thoroughly compared with DA in terms of solution quality, computational time, convergence rate, and number of failures in obtaining pre-specified targets. Both source codes are implemented in the C++ and run under the same conditions.

*The value of objective function after equal run times*. The two algorithms are first compared based on the value of objective function returned after a prespecified run time. To decide about the prespecified run time, the proposed DA by Braekers, Caris [17] with the recommended termination criteria is applied to each instance and the run time to reach the termination condition is recorded. This is repeated 21 times (for every instance) and the average run time calculated from this exercise is recorded and used as the prespecified run time. Then, both algorithms are run for the prespecified run time on each instance for 21 times and the results are presented in Table 2. This examination is carried out to assess the proposed method’s ability in terms of solution quality under identical conditions, in comparison with the DA algorithm.

**Table 2:** Quality of solutions in LD-DA and DA after a pre-specified run time on the standard DARP instances of [14].

Instance	CPU	DA [17]					LD-DA					*Gap* (%)				
		Best	Worst	Mean	SD.	Median	Best	Worst	Mean	SD.	Median	Best	Worst	Mean	SD.	Median
pr01	16.09	190.02	204.78	191.09	3.47	190.02	190.02	190.02	190.02	0.00	190.02	0.00	-7.21	-0.56	-100.0	0.00
pr02	40.30	301.34	351.43	319.14	19.64	301.34	301.16	305.34	302.68	1.24	302.75	-0.06	-13.12	-5.16	-93.6	0.47
pr03	46.51	532.10	622.91	572.12	32.68	580.94	529.15	548.93	537.13	6.08	536.00	-0.55	-11.88	-6.12	-81.4	-7.74
pr04	70.90	585.57	630.67	608.12	22.55	585.57	576.36	590.79	580.86	3.58	577.64	-1.57	-6.32	-4.48	-84.1	-1.35
pr05	78.51	631.27	734.63	678.57	28.56	679.98	636.61	651.82	642.23	4.01	641.21	0.85	-11.27	-5.36	-85.9	-5.70
pr06	100.5	800.12	908.97	862.36	26.62	873.09	796.56	818.31	809.88	5.20	807.11	-0.44	-9.97	-6.09	-80.4	-7.56
pr07	21.83	292.23	292.23	292.23	0.00	292.23	291.71	294.85	293.00	1.30	291.71	-0.18	0.89	0.26	…^1^	-0.18
pr08	41.79	488.60	497.72	492.35	2.65	491.01	491.74	512.13	501.70	6.85	502.29	0.64	2.89	1.90	158.4	2.30
pr09	65.36	661.30	693.44	670.94	8.94	678.19	661.70	697.70	683.27	9.63	692.62	0.06	0.61	1.84	7.7	2.13
pr10	101.8	857.41	876.71	865.55	4.77	864.83	867.92	901.83	886.42	9.02	886.32	1.23	2.87	2.41	89.1	2.49
pr11	22.90	164.46	175.22	165.43	2.98	164.46	164.46	174.64	165.46	2.49	164.53	0.00	-0.33	0.02	-16.4	0.04
pr12	50.53	296.18	341.40	310.29	15.82	298.77	295.27	306.47	297.99	2.90	296.81	-0.31	-10.23	-3.96	-81.6	-0.66
pr13	72.24	488.61	582.08	525.62	29.52	535.28	485.43	501.16	493.70	3.71	492.86	-0.65	-13.90	-6.07	-87.4	-7.92
pr14	105.4	534.52	637.88	568.42	31.17	567.91	535.23	550.47	542.17	4.18	543.50	0.13	-13.70	-4.62	-86.5	-4.30
pr15	146.8	578.42	713.46	623.51	34.06	631.46	583.75	601.45	590.38	5.08	596.61	0.92	-15.70	-5.31	-85.0	-5.52
pr16	167.4	739.29	841.66	772.62	29.45	777.61	742.68	771.84	757.24	7.50	757.23	0.46	-8.30	-1.99	-74.5	-2.62
pr17	29.83	249.33	309.16	260.12	19.72	249.33	248.21	265.31	251.98	4.33	252.39	-0.45	-14.18	-3.13	-78.0	1.23
pr18	70.82	458.73	528.69	484.59	25.85	465.98	463.36	475.21	467.76	2.78	470.56	1.01	-10.12	-3.47	-89.2	0.98
pr19	133.1	597.34	686.40	609.70	22.84	602.45	603.72	616.70	610.79	3.56	605.76	1.07	-10.15	0.18	-84.4	0.55
pr20	156.5	795.49	816.84	804.01	5.96	802.89	795.49	825.55	804.01	5.96	804.49	0.01	1.07	0.00	0.0	0.09
**Avg.**	**76.98**	**512.12**	**572.31**	**533.84**	**18.36**	**531.67**	**513.03**	**530.03**	**520.43**	**4.47**	**520.54**	**0.11**	**-7.40**	**-2.49**	**-50.20**	**-1.67**

1- Standard Deviation related to this instance for Braekers DA was equal to zero, so we excluded it from gap calculations.

Table 2 displays the prespecified CPU time followed by the best and worst solutions, and the mean, standard deviation, and median of 21 trials obtained from both algorithms. The gaps between the reported values are presented in percentage metrics, where returned solutions by Braekers, Caris [17] were taken as the baseline for comparison. The findings indicate that, on average, the proposed LD-DA algorithm exhibits superior performance over Braekers and Caris (16) DA algorithm in relation to the mean, median, and worst solutions, with gaps of -2.49 percent, -1.67 percent, and -7.40 percent, respectively. However, the proposed LD-DA algorithm slightly lags behind in terms of the best solution obtained, with an average difference of 0.11 percent compared to the DA algorithm.

We use the Wilcoxon rank-sum test [62] to examine if the performance of the proposed method is statistically different from the DA. The Wilcoxon rank-sum test compares the algorithms for each instance using the results of 21 runs and determines if the performance of one algorithm is statistically better than the other, or if there is no statistical difference between the performances (tie situation). The results are reported in Table 3. Based on the Wilcoxon test, LD-DA outperforms DA with 9 wins (45 percent), 6 ties (30 percent), and 5 losses (25 percent). The assessments underscore the capability of the proposed LD_DA method to yield high-quality solutions. In these identical conditions (with the same computational time), LD_DA demonstrates a more consistent and reliable performance, presenting a higher likelihood of producing solutions with superior quality.

**Table 3:** Results of rank sum Wilcoxon test (same CPU time considered for both algorithms).

Instance	P-value	DA [17]	LD-DA	Tie situation
pr01	0.00		✓	
pr02	0.16			✓
pr03	0.01		✓	
pr04	0.00		✓	
pr05	0.00		✓	
pr06	0.00		✓	
pr07	0.78			✓
pr08	0.00	✓		
pr09	0.00	✓		
pr10	0.00	✓		
pr11	0.00	✓		
pr12	0.01		✓	
pr13	0.04		✓	
pr14	0.05		✓	
pr15	0.01		✓	
pr16	0.40			✓
pr17	0.61			✓
pr18	0.61			✓
pr19	0.00	✓		
pr20	0.13			✓
Sum		5	9	6

*Comparing computational time to achieve a prespecified objective value*. The second experiment compares the time taken by both algorithms to reach pre-specified objective targets. The target values are set as the highest objective value obtained by both algorithms from the results reported in Table 2 to ensure the target is achievable for both algorithms. Both algorithms are run 21 times to each instance, and the results are presented in Table 4. Overall, the results show superiority of the LD_DA in terms of computational time in comparison with the DA. The last four columns of the Table 4 report the ratios of the run time from the DA algorithm over the run time from the LD_DA. As shown, LD-DA has a significantly lower run time across all the instances. On average, the proposed method is 3.88 times faster than DA in reaching the identical pre-specified quality targets.

**Table 4:** Run times of LD-DA and DA in converging to pre-specified targets on the standard DARP instance of [14].

Instance	Set Target	DA [17]					LD-DA					Ratio			
		Min	Max	Mean	SD	Median	Min	Max	Mean	SD	Median	Min	Max	Mean	Median
pr01	204.78	0.33	0.33	0.33	0.31	0.33	0.08	0.10	0.08	0.07	0.08	4.1	3.5	4.0	4.12
pr02	351.43	0.95	1.70	1.01	1.10	0.96	0.28	0.31	0.28	0.28	0.28	3.4	5.5	3.6	3.44
pr03	622.91	1.37	1.86	1.41	1.47	1.39	0.49	0.50	0.49	0.48	0.49	2.8	3.7	2.9	2.85
pr04	630.67	3.22	4.14	3.38	3.37	3.31	1.17	1.17	1.17	1.14	1.17	2.8	3.5	2.9	2.83
pr05	734.63	3.84	4.24	3.94	3.91	3.90	1.95	2.28	2.06	2.02	1.99	2.0	1.9	1.9	1.96
pr06	908.97	5.03	9.25	6.14	6.12	5.80	2.14	2.24	2.18	2.12	2.17	2.4	4.1	2.8	2.67
pr07	294.84	0.49	6.96	1.33	1.97	0.76	0.25	0.29	0.27	0.18	0.26	2.0	23.9	5.0	2.91
pr08	518.12	1.52	1.85	1.67	1.54	1.68	0.99	4.14	1.51	1.59	1.21	1.5	0.4	1.1	1.38
pr09	697.69	2.88	5.84	4.16	3.45	4.07	2.71	6.94	4.15	3.36	4.11	1.1	0.8	1.0	0.99
pr10	901.82	4.77	6.34	5.28	4.96	5.28	3.65	8.16	5.25	4.96	4.61	1.3	0.8	1.0	1.15
pr11	175.22	0.72	0.81	0.75	0.69	0.75	0.16	0.18	0.17	0.14	0.17	4.5	4.5	4.3	4.40
pr12	341.4	1.79	2.02	1.82	1.83	1.80	0.32	0.49	0.36	0.35	0.33	5.6	4.1	5.0	5.44
pr13	582.08	2.99	8.36	3.29	4.11	3.03	0.45	0.47	0.45	0.44	0.45	6.7	17.8	7.3	6.73
`pr14	637.88	4.81	4.97	4.87	4.81	4.88	0.81	0.83	0.82	0.79	0.82	5.9	6.0	5.9	5.96
pr15	713.46	8.29	12.95	8.98	9.22	8.58	2.04	2.07	2.06	1.98	2.06	4.1	6.3	4.4	4.17
pr16	841.66	9.95	17.96	13.90	11.34	13.94	1.73	1.79	1.75	1.57	1.74	5.8	10.0	8.0	8.01
pr17	309.16	1.03	1.17	1.05	1.05	1.04	0.19	0.20	0.20	0.19	0.20	5.3	5.7	5.2	5.08
pr18	528.69	3.60	46.18	5.85	12.82	3.65	0.82	0.88	0.84	0.80	0.82	4.4	52.2	7.0	4.42
pr19	686.4	7.23	7.81	7.30	7.24	7.25	2.63	2.68	2.66	2.50	2.66	2.7	2.9	2.7	2.72
pr20	834.63	10.53	17.02	12.52	10.62	11.73	5.49	10.23	6.90	6.01	6.29	1.9	1.7	1.8	1.86
Avg.	575.82	3.76	8.08	4.44	4.59	4.20	1.41	2.29	1.68	1.54	1.59	3.51	7.96	3.88	3.65

*Comparing the convergence rate*. The two algorithms are also compared with respect to their convergence rate. To do so, the convergence graphs related to the median run of both algorithms are compared. Each algorithm is run on each instance for the considered computational time in the Section ‎5.2.3.1‎5.2.3.1 (second column, Table 2). All changes related to the best generated solution for each instance is recorded and the corresponding convergence graph is plotted. Then the convergence graphs corresponding to the median runs of each instance are compared.

**Fig 1:**
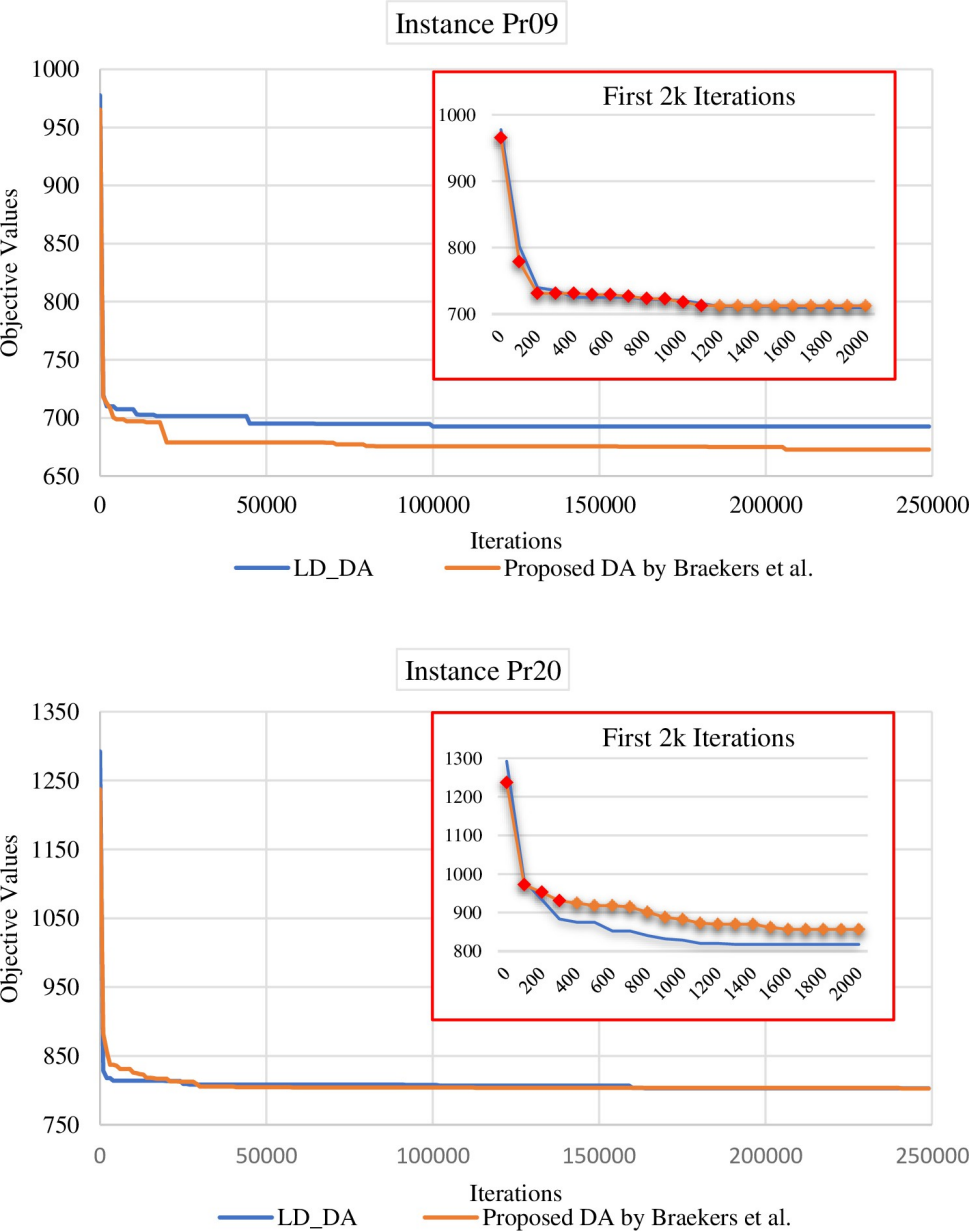
Convergence graphs related to the instances Pr09 and Pr20 for the proposed LD-DA and the DA proposed by Braekers, Caris [17].

Fig 1 shows the convergence graphs for instances Pr09 and Pr20. As it can be seen both algorithms show almost the same behaviour in sharply decreasing at the early stages of the optimisation process. However, the proposed DA by Braekers, Caris [17] starts with infeasible initial solution and continues in the infeasible region until a feasible solution is identified. The transition into feasibility takes place during iteration 1154 in the case of instance PR09 and iteration 312 for instance PR20. This implies that the Braekers, Caris [17] DA approach may not ensure a feasible solution if terminated prematurely during the optimisation process. In contrast, the proposed LD-DA method exclusively explores the feasible search space, ensuring the return of a feasible solution regardless of the termination point. This aspect underscores the dependability of LD-DA in dynamic scenarios, where obtaining high-quality feasible solutions within limited run times is crucial.

*Number of failures*. In the fourth experiment, the number of failures in reaching prespecified targets is compared. In this experiment, the median objective values obtained in the experiment section ‎5.2.3.1 (Table 2, columns 13 and 17) are set as objective value targets for both algorithms to achieve. Then, each algorithm is run 21 times for each instance. The algorithm is terminated if the target is achieved, or the run time exceeds 5 minutes. If the algorithm cannot reach the target objective within 5 minutes, the case is recorded as a failure. In this experiment, we are after comparing methods’ ability of escaping from local optima.

The results are presented in Table 5. The medians obtained in the first experiment are different across the methods, therefore this experiment is repeated twice. Table 5(A) shows the results for targets obtained from the LD-DA medians and Table 5(B) shows the results for the targets based on the DA medians. Comparing the number of failures across the tables one can observe that the performance of a method is better when the targets are set based on the median of its own. A comparative analysis of performance among the methods reveals a notably reduced frequency of failures for LD-DA. Specifically, DA falls short of achieving the targets defined by the proposed LD-DA method on 151 occasions, and similarly, it does not meet its own set targets 128 times. In contrast, the proposed LD-DA method experiences 64 and 94 failures respectively. This observation could potentially suggest a stronger exploration ability of the proposed LD_DA method and its capacity to avoid becoming trapped in local optima.

**Table 5:** Number of algorithms’ failures in reaching set objective targets.

Instance	Number of Failures				Difference
	[17]		LD-DA		
pr01	2		0		-2
pr02	10		5		-5
pr03	13		3		-10
pr04	11		1		-10
pr05	17		8		-9
pr06	16		0		-16
pr07	0		0		0
pr08	0		10		10
pr09	2		4		2
pr10	0		3		3
pr11	2		3		1
pr12	11		5		-6
pr13	13		2		-11
pr14	11		6		-5
pr15	13		2		-11
pr16	11		2		-9
pr17	5		3		-2
pr18	9		1		-8
pr19	2		0		-2
pr20	3		6		3
Sum		151	64		-87
(a) target: obtained medians by proposed DA					
Instance	Number of Failures				Difference
	[17]			LD-DA	
pr01	2			0	-2
pr02	10			5	-5
pr03	10			0	-10
pr04	10			1	-9
pr05	10			0	-10
pr06	6			0	-6
pr07	0			0	0
pr08	2			16	14
pr09	4			15	11
pr10	3			18	15
pr11	2			3	1
pr12	10			5	-5
pr13	10			0	-10
pr14	10			0	-10
pr15	10			0	-10
pr16	10			0	-10
pr17	5			3	-2
pr18	9			8	-1
pr19	2			12	10
pr20	3			8	5
Sum	128			94	-34
(b) target: obtained medians by DA [17]					

In summary, the comparison between LD-DA and DA reveals that both algorithms are capable of delivering quality solutions. Nevertheless, a more detailed analysis unveils several advantages held by LD-DA over DA. Despite having similar convergence rates, LD-DA achieves pre-specified objective targets 3.5 times faster than DA. Moreover, LD-DA demonstrates higher reliability in achieving these targets, evident by fewer failures in comparison to DA. Additionally, the convergence graph highlights LD-DA’s ability to provide feasible solutions at any point during the optimisation process, a capability that DA lacks. Overall, LD-DA outperforms DA across various aspects within identical computational conditions, even though both algorithms are capable of providing high-quality solutions.

### The effect of LD-threshold adjusting

The main contribution of the proposed algorithm is adopting new threshold adjusting approach for deterministic algorithms. Therefore, in this sub-section, the effectiveness of the LD-threshold adjusting for deterministic annealing in solving DARP is examined. To do so, the performance of LD-threshold mechanism is compared with the existing accepting threshold mechanism adopted by Braekers, Caris [17]. To make a fair comparison, both adjusting mechanisms are implemented on the DA method proposed by Braekers, Caris [17]. Both mechanisms are compared from two angles: first, quality of returned solutions after the same computational time, and second, the required computational time for reaching prespecified objective values.

#### The value of objective function after equal run times

This section compares the two mechanisms for adjusting thresholds and evaluates them based on their objective functions after a prespecified computational time. The prespecified run time for each instance is equal to what is considered in the experiment in the Section ‎5.2.3.1 (Table 2 column 2). Both algorithms are run on each instance for 21 times and the results in terms of quality of solutions (value of objective function) are presented in Table 6.

**Table 6:** Efficiency of LD-threshold on solutions’ quality on 20 standard DARP instance of [14].

Instance	CPU	DA [17]					DA [17] + LD-threshold					*Gap* (%)				
		Best	Worst	Mean	SD.	Median	Best	Worst	Mean	SD.	Median	Best	Worst	Mean	SD.	Median
pr01	16.09	190.02	204.78	191.09	3.47	190.02	190.02	190.02	190.02	0	190.02	0.00	-7.21	-0.56	-100.00	0.00
pr02	40.30	301.34	351.43	319.14	19.64	301.34	301.34	348.11	312.45	9.81	301.34	0.00	-0.94	-2.10	-50.05	0.00
pr03	46.51	532.1	622.91	572.12	32.68	580.94	533.26	612.39	551.81	11.48	536.44	0.22	-1.69	-3.55	-64.87	-7.66
pr04	70.90	585.57	630.67	608.12	22.55	585.57	581.69	620.09	594.43	9.19	587.93	-0.66	-1.68	-2.25	-59.25	0.40
pr05	78.51	631.27	734.63	678.57	28.56	679.98	632.66	707.51	647.41	23.66	659.13	0.22	-3.69	-4.59	-17.16	-3.07
pr06	100.5	800.12	908.97	862.36	26.62	873.09	801.97	882.43	842.14	14.75	839.27	0.23	-2.92	-2.34	-44.59	-3.87
pr07	21.83	292.23	292.23	292.23	0	292.23	292.23	292.23	292.23	0	292.23	0.00	0.00	0.00	0.00	0.00
pr08	41.79	488.6	497.72	492.35	2.65	491.01	487.84	494.92	491.85	2.39	492.78	-0.16	-0.56	-0.10	-9.81	0.36
pr09	65.36	661.3	693.44	670.94	8.94	678.19	663.85	676.59	668.98	3.17	669.17	0.39	-2.43	-0.29	-64.54	-1.33
pr10	101.8	857.41	876.71	865.55	4.77	864.83	861.11	883.71	868.92	5.4	863.83	0.43	0.80	0.39	13.21	-0.12
pr11	22.90	164.46	175.22	165.43	2.98	164.46	164.46	184.63	165.44	4.4	164.46	0.00	5.37	0.01	47.65	0.00
pr12	50.53	296.18	341.4	310.29	15.82	298.77	296.37	338.35	305.49	11.73	302.37	0.06	-0.89	-1.55	-25.85	1.20
pr13	72.24	488.61	582.08	525.62	29.52	535.28	489.05	581.66	518.58	16.28	493.53	0.09	-0.07	-1.34	-44.85	-7.80
pr14	105.4	534.52	637.88	568.42	31.17	567.91	534.81	613.44	567.49	17.53	564.78	0.05	-3.83	-0.16	-43.76	-0.55
pr15	146.8	578.42	713.46	623.51	34.06	631.46	579.30	698.3	619.33	19.47	636.87	0.15	-2.12	-0.67	-42.84	0.86
pr16	167.4	739.29	841.66	772.62	29.45	777.61	740.39	829.17	759.83	20.53	761.54	0.15	-1.48	-1.66	-30.29	-2.07
pr17	29.83	249.33	309.16	260.12	19.72	249.33	249.33	294.03	259.05	11.21	249.33	0.00	-4.89	-0.41	-43.15	0.00
pr18	70.82	458.73	528.69	484.59	25.85	465.98	459.13	517.89	481.27	17.34	462.55	0.09	-2.04	-0.69	-32.92	-0.74
pr19	133.1	597.34	686.4	609.7	22.84	602.45	598.11	675.87	606.79	11.65	607.46	0.13	-1.53	-0.48	-48.99	0.83
pr20	156.5	795.49	816.84	804.01	5.96	802.89	797.14	817.15	805.21	5.05	805.8	0.21	0.04	0.15	-15.27	0.36
**Avg.**	**76.98**	**512.12**	**572.31**	**533.84**	**18.36**	**531.67**	**512.70**	**562.92**	**527.44**	**10.75**	**524.04**	**0.08**	**-1.59**	**-1.11**	**-33.87**	**-1.16**

Table 6 presents the prespecified CPU time, followed by the best and worst solutions, and the mean, standard deviation and median of the 21 trials returned from the two algorithms. The gaps between the reported values are presented in subsequent columns. The gaps are calculated in percentage metrics where the existing accepting threshold adjusting mechanism is considered as the baseline of comparison.

According to the results, the mean, median and worst solutions returned by the LD-threshold mechanism are statistically superior compared to their counterparts returned by the existing accepting threshold mechanism. On average, the gaps for worst, mean, and median are -1.59 percent, -1.11 percent, and -1.16 percent, respectively. However, in terms of the best returned solution, the LD-threshold slightly under performs existing accepting threshold mechanism, as the best solution from the LD-threshold is on average 0.08 percent worse compared to the ones returned by original DA.

The Wilcoxon rank-sum test is used to investigate if the differences observed between the performance of LD-threshold and existing accepting threshold are statistically significant. The Wilcoxon rank-sum test compares the obtained results from 21 runs of each threshold mechanism on every instance to signifies if there is any statistical difference the obtained results by each algorithm. The Wilcoxon test results are reported in Table 7. The results indicate a statistically significant superior performance of the LD-threshold in 5 instances (25 percent of the tested cases). Conversely, there is no statistically significant difference in performance in 14 instances (70 percent), and a performance disadvantage is observed in 1 instance (5 percent). The primary objective of this experiment is to assess the effectiveness of the LD-threshold adjusting approach in steering clear of local optima during the optimisation process and generating high-quality solutions. The results obtained from this experiment, as presented in Tables 6 and 7, demonstrate that the LD-threshold adjusting approach excels not only in delivering high-quality solutions for various instances but also exhibits a marginal superiority over the existing threshold mechanism. The LD-threshold approach manages to reduce the objective function value by an average of 1.59 percent and statistically outperforms the current mechanism in 25 percent of cases.

**Table 7:** Wilcoxon rank-sum test for threshold adjusting mechanism.

Instance	P-value	DA [17]	DA [17] + LD-threshold	Tie situation
pr01	0.24			✓
pr02	0.50			✓
pr03	0.41			✓
pr04	0.01		✓	
pr05	0.01		✓	
pr06	0.00		✓	
pr07	1.00			✓
pr08	0.74			✓
pr09	0.01		✓	
pr10	0.01	✓		
pr11	0.87			✓
pr12	0.39			✓
pr13	0.43			✓
pr14	0.93			✓
pr15	0.52			✓
pr16	0.03		✓	
pr17	0.89			✓
pr18	0.90			✓
pr19	0.54			✓
pr20	0.90			✓
Sum		1	5	14

#### Comparing computational time to achieve a prespecified objective value

The two threshold adjusting mechanisms are compared based on the required computational time. This experiment compares required run time (includes initialisation and optimisation) for both threshold mechanisms to reach prespecified objective values. The target for each instance is set as the worst objective value across 21 runs of both mechanisms reported in Table 4. This ensures the algorithms can always reach the targets. Each algorithm is then applied 21 times to each instance and the required run times are reported in Table 8.

**Table 8:** Efficiency assessment LD-threshold on run time on 20 standard DARP instance of [14].

Instance	Set Target	DA [17]					DA [17] + LD-threshold					Ratio			
		Min	Max	Mean	SD	Median	Min	Max	Mean	SD	Median	Min	Max	Mean	Median
pr01	204.78	0.33	0.33	0.33	0.31	0.33	0.06	0.07	0.06	0.04	0.06	5.50	4.71	5.50	5.50
pr02	351.43	0.95	1.7	1.01	1.1	0.96	0.18	0.44	0.33	0.22	0.33	5.28	3.86	3.06	2.91
pr03	622.91	1.37	1.86	1.41	1.47	1.39	0.44	0.59	0.52	0.31	0.58	3.11	3.15	2.71	2.40
pr04	630.67	3.22	4.14	3.38	3.37	3.31	1.21	4.88	2.64	2.25	2.54	2.66	0.85	1.28	1.30
pr05	734.63	3.84	4.24	3.94	3.91	3.9	2.05	3.43	2.63	2.07	2.71	1.87	1.24	1.50	1.44
pr06	908.97	5.03	9.25	6.14	6.12	5.8	3.81	6.43	3.83	1.81	3.48	1.32	1.44	1.60	1.67
pr07	292.23	0.49	6.96	1.33	1.97	0.76	0.27	1.28	0.91	1.18	0.68	1.81	5.44	1.46	1.12
pr08	497.72	1.52	1.85	1.67	1.54	1.68	0.76	1.63	1.48	1.15	1.14	2.00	1.13	1.13	1.47
pr09	693.44	2.88	5.84	4.16	3.45	4.07	2.42	5.73	4.45	3.31	4.32	1.19	1.02	0.93	0.94
pr10	883.71	4.77	6.34	5.28	4.96	5.28	3.34	7.83	5.48	4.97	5.37	1.43	0.81	0.96	0.98
pr11	184.63	0.72	0.81	0.75	0.69	0.75	0.19	0.31	0.24	0.11	0.23	3.79	2.61	3.13	3.26
pr12	341.4	1.79	2.02	1.82	1.83	1.8	0.31	0.72	0.55	0.38	0.49	5.77	2.81	3.31	3.67
pr13	582.08	2.99	8.36	3.29	4.11	3.03	0.61	0.96	0.72	0.31	0.74	4.90	8.71	4.57	4.09
`pr14	637.88	4.81	4.97	4.87	4.81	4.88	1.18	2.41	1.58	0.96	1.67	4.08	2.06	3.08	2.92
pr15	713.46	8.29	12.95	8.98	9.22	8.58	3.34	8.71	6.23	6.07	5.78	2.48	1.49	1.44	1.48
pr16	841.66	9.95	17.96	13.9	11.34	13.94	4.36	23.52	8.75	7.79	6.78	2.28	0.76	1.59	2.06
pr17	309.16	1.03	1.17	1.05	1.05	1.04	0.31	0.58	0.47	0.03	0.46	3.32	2.02	2.23	2.26
pr18	528.69	3.60	46.18	5.85	12.82	3.65	1.24	9.64	3.42	7.06	3.87	2.90	4.79	1.71	0.94
pr19	686.4	7.23	7.81	7.3	7.24	7.25	4.41	8.21	5.81	2.7	5.93	1.64	0.95	1.26	1.22
pr20	817.15	10.53	17.02	12.52	10.62	11.73	7.73	12.47	10.63	3.07	10.45	1.36	1.36	1.18	1.12
Avg.	204.78	3.77	8.09	4.45	4.60	4.21	1.91	4.99	3.04	2.29	2.88	2.94	2.56	2.18	2.14

The reported ratios in the last columns are equal to ratios of the existing accepting threshold mechanism run time over the required run time for LD-threshold mechanism. Based on the results, the proposed LD-threshold mechanism is significantly faster in reaching set targets across all the instances. On average, the DA-threshold is 2.18 times faster than the original DA threshold mechanism.

Obtained results by LD-threshold mechanism suggests that the LD mechanism can help DA algorithms to improve in terms of quality of returned solutions and run time. Reported results in Table 6 showed that the same DA algorithm with LD-threshold is more likely to end with better objective values in comparison with the existing accepting threshold adjusting mechanism where the mean and median of obtained objective values are reduced on average by 1.11 percent and 1.16 percent, respectively. In addition, obtained results from Table 8 demonstrated that it is more than 2 times faster in reaching the pre-specified objective values from all compared angles: minimum, maximum, average, and median of required run time. Overall, the results of the examination in this sub-section indicate that the newly proposed LD-threshold adjusting approach outperform the existing threshold adjusting approach. By adopting the LD-threshold adjusting approach, a slight superiority is observed in terms of the mean, median, and standard deviation of the obtained solutions in terms of quality. These results highlight the LD-threshold approach’s superiority not only in terms of solution quality but also in terms of computational efficiency. The adoption of LD-threshold adjustment effectively accelerates the process of obtaining quality targets, reducing the process time by more than two times.

## Conclusion

The Dial-a-Ride Problem (DARP) is a distinct version of the vehicle routing problem that centres around passenger transport. It finds use in various scenarios, such as assisting passengers with specific needs, serving specific locations like airports, and addressing public transport needs in low-demand areas. However, due to the NP-hard nature of DARPs, finding efficient solutions within practical computational time is difficult, restricting its applicability to smaller regions and fewer requests. As problem complexity grows, implementing DARP becomes more challenging. In our research, we have introduced a more efficient method to address larger-scale DARP applications. This method contributes to the field in two ways. Firstly, it introduces the Linearly Decreasing-Deterministic Annealing (LD-DA) algorithm, notable for its innovative threshold adjustment mechanism. This mechanism enables the rapid attainment of high-quality DARP solutions while significantly reducing computational time. Secondly, the LD-DA algorithm possesses the ability to swiftly generate feasible solutions right after initialisation. Access to feasible solutions at any time after initialisation, even when achieving a high-quality solution within time constraints is not feasible, makes the proposed method practical for real-time and time-sensitive applications. This facilitates replacing large-scale public transport systems in real-world scenarios with DARP solutions, ensuring that practical operational plans are always accessible.

The initialisation process generates a feasible solution, and throughout the optimisation process, it consistently explores the feasible solution space. This ensures the availability of a feasible solution at any given termination point. The local search operators are meticulously designed to enhance the process of selecting requests to be serviced by the vehicles in a cost-effective manner. Unlike existing operators that heavily rely on the random reinsertion of requests, our proposed LD-DA method selects requests based on associated travel costs for their inclusion in the current routes. The third enhancement emerges from the adoption of a distinct threshold adjustment scheme. This threshold plays a crucial role in the local search by determining the acceptance of non-improved solutions, aiding in escaping local optima. In this study, the adopted mechanism imposes a linear constraint on the jump size as the algorithm converges towards optima. Overall, the proposed method demonstrates comparable performance with other state-of-the-art methods in the literature in terms of the quality of obtained solutions, while significantly reducing the required computational time for achieving high-quality solutions. This renders it a more reliable method for larger-scale problems, such as real-world instances, in obtaining high-quality solutions within a practical timeframe. Furthermore, its ability to return feasible solutions at any point after initialisation makes it adaptable to dynamic settings, a highly sought-after feature in real-world scenarios.

The efficiency of the proposed method is evaluated against the most advanced and efficient methods in the literature, on a widely used set of benchmarks for standard DARP. Overall, the proposed method is successful in obtaining comparable quality of solution to the best-known solution (with the gap of only 0.91 percent). The LD-DA also achieved equal or better quality of solutions compared to BKS in 7 instances. Furthermore, the proposed LD-DA shows a competitive performance compared to other state of the are algorithms, while it saves considerable amount of computational time in comparison with the other techniques. Specifically, the LD-DA is almost 3 times faster than Pure ALNS, 10 times faster than Best configuration ALNS, and 28 times faster than ELN. Moreover, a comprehensive series of experiments is conducted to allow for a fair comparison. The performance of LD-DA is assessed from three different angles in comparison with the DA algorithm proposed by Braekers, Caris [17]. First, the objective functions after identical run times are compared, and the results indicate that LD-DA shows better performance in comparison to DA and reduces mean and median of objective values by 2.49 percent and 1.67 percent on average, respectively. The Wilcoxon rank-sum test indicates that the improvement offered by LD-DA is statistically significant. Secondly, the run time to achieve prespecified targets are compared and the results indicated that LD-DA can reach the targets on an average 3.5 times faster. Finally, number of failures in achieving a prespecified target within a relatively long run time is compared, and the results once again indicates superior performance of LD-DA over DA.

The main contribution of the proposed method is adopting the LD-threshold mechanism. The contribution of LD-threshold is compared with the existing accepting threshold mechanism by comparing the quality of solutions after identical run times and required run time to achieve identical objective values. The results indicated that implementing the LD-threshold mechanism can reduce the mean and median by 1.11 percent and 1.16 percent, respectively. The Wilcoxon rank-sum test indicated that the improvements from adopting LD-threshold are statistically significant. Comparing the required run time to obtain set objective values, LD-thresholding can offer considerable reduction in computational time i.e., nearly half of that required by existing accepting threshold mechanisms.

While the proposed method is computationally efficient, it is still restricted to standard DARPs in the current form. A significant research direction for future exploration involves adapting the proposed algorithms to encompass alternative objective functions, such as minimising passenger inconvenience or addressing environmental impacts of the transportation system. Additionally, incorporating more real-life problem characteristics, such as considering passenger and vehicle’s heterogeneity, real-time considerations, driver breaks, time-dependent travel times, and real-time passenger requests, would enhance the method’s practical applicability. This broader incorporation of elements would undoubtedly enhance the method’s versatility, making it an even more valuable tool for handling a wider array of real-world transportation challenges.

## Supporting information

S1 Appendix (DOCX)

S2 Appendix (DOCX)
