# Supplementary material for: A linearly decreasing deterministic annealing algorithm for the multi-vehicle dial-a-ride problem
Source: PLoS One. 2024 Feb 8;19(2):e0292683. doi: 10.1371/journal.pone.0292683 (PMC10852268; doi:10.1371/journal.pone.0292683)
Supplement: S1 Appendix — (DOCX) [file pone.0292683.s001.docx]

# Appendix A

As discussed in section ‎4, the proposed DA algorithm contains five user defined parameters. These parameters are number of iterations ($n_{iter}$), maximum threshold ($T_{max}$), minimum threshold ($T_{min}$), threshold reduction parameter ($T_{reduction}$), and restart iteration parameter ($n_{restart}$ ). Sensitivity analysis is carried out to decide about the values of $n_{restart}$,$T_{reduction}$ ,$coef_{t,max}$ and $coef_{t,min}$. The parameter $n_{iter}$ is designated to determines the optimising process of the algorithm. In the DA algorithms, non-improved solutions are accepted if objective value’s deterioration is less than the acceptance threshold. In the proposed LD-DA the acceptance threshold is limited to $T_{max}$ and $T_{min}$ during optimising process. In every iteration that $x_{best}$ (best so far solution) remains fixed, the algorithm reduces the value of acceptance threshold according to the $T_{reduction}$. The $n_{restart}$ is defined to help algorithm to escape from local optima, by resetting value of accepting threshold to the maximum possible threshold ($T_{max}$), if there is no improvement in $x_{best}$ after $n_{restart}$ iterations.

In the sensitivity analysis the initial values of $n_{iter}$, $n_{restart}$, and $T_{reduction}$ are set to 100,000, 500 and 500, respectively. The other two parameters of $T_{max}$ and $T_{min}$ are defined as functions of the average arc travel cost: $T_{max} =Coef_{t,max}\times average arcs^{'} cost$ and $T_{min}=Coef_{t,min}\times T_{max}$. In these equations, $Coef_{t,max}$ and $Coef_{t,min}$ are initially set to 1.5 and 0.5 respectively. The sensitivity analysis is tested on 4 instances from the Cordeau and Laporte (2003) benchmark. To cover a variety of instance sizes, two of the instances are selected from small instances (Pr03 and Pr13) and the other two from large instances (Pr06, and Pr16).

Since in conducted experiments proposed DA terminates at the same point with the compared method, we did not carry out sensitivity analysis for setting value of $n_{iter}$ and it is set to initial value during sensitivity analysis for other parameters. However, different values are considered for the rest of parameters and their values are set one by one. First, value of $n_{restart}$ is set, where for each considered value 5 tests carried out, while the rest of parameters were set to their initial values. Then, the gap between average of results and best-found solution by Parragh et al. (2013) is calculated. The value with the minimum gap is set as the fixed value of $n_{restart}$. The same procedure was taken for the rest of parameters, while already investigated parameters were set to their fixed values and the rest were set to their initial values. Based on the observations, values of $Coef_{t,max}$, $Coef_{t,min}$, $T_{reduction}$, and $n_{restart}$ parameters are set to 2, 0.2, 300, and 300, respectively. Table 9 reports considered values and resulting gaps for each parameter.

Table 9 - Sensitivity Analysis for determining values of user-defined parameters of the proposed LD_DA.

| $n_{restart}$ | Values | 100 | 200 | 300 | 400 | 500 | 600 | 700 | 800 | 900 | 1000 | 2000 |
| --- | --- | --- | --- | --- | --- | --- | --- | --- | --- | --- | --- | --- |
|  | Avg Gap (%) | 1.04 | 1.02 | 0.97 | 0.99 | 1.01 | 1.01 | 1.07 | 1.19 | 1.35 | 1.53 | 2.22 |
| $T_{reduction}$ | Values | 100 | 200 | 300 | 400 | 500 | 600 | 700 | 800 | 900 | 1000 | 2000 |
|  | Avg Gap (%) | 1.12 | 1.03 | 0.95 | 0.96 | 0.98 | 1.00 | 1.05 | 1.06 | 1.06 | 1.11 | 1.52 |
| $Coef_{t,max}$ | Values | 1.0 | 1.2 | 1.4 | 1.6 | 1.8 | 2.0 | 2.2 | 2.4 | 2.6 | 2.8 | 3.0 |
|  | Avg Gap (%) | 0.98 | 0.96 | 0.96 | 0.95 | 0.89 | 0.87 | 0.92 | 1.02 | 1.31 | 1.60 | 2.34 |
| $Coef_{t,min}$ | Values | 0.0 | 0.2 | 0.4 | 0.6 | 0.8 | 1.0 |  |  |  |  |  |
|  | Avg Gap (%) | 0.92 | 0.84 | 1.03 | 1.19 | 1.33 | 1.45 |  |  |  |  |  |
