# Supplementary material for: A linearly decreasing deterministic annealing algorithm for the multi-vehicle dial-a-ride problem
Source: PLoS One. 2024 Feb 8;19(2):e0292683. doi: 10.1371/journal.pone.0292683 (PMC10852268; doi:10.1371/journal.pone.0292683)
Supplement: S2 Appendix — (DOCX) [file pone.0292683.s002.docx]

# Appendix B

This section presents the comprehensive and detailed comparison between the LD-DA and the state of art algorithms, ELS and ALNS. Before comparing results, we have to set the LD-DA termination condition. In Table 10, different termination’s points are compared. It is showed that performance of the LD-DA remained almost the same after 50,000 iterations, which we have used for the rest of analysis in this Section and Section ‎5.2.2.

Table 10- Comparing performance of the proposed LD_DA with different termination’s point.

| instance | termination:25k iterations | | |  | termination:50k iterations | | |  | termination:75k iterations | | |  | termination:100k iterations | | |
| --- | --- | --- | --- | --- | --- | --- | --- | --- | --- | --- | --- | --- | --- | --- | --- |
|  | Best | Avg | CPU |  | Best | Avg | CPU |  | Best | Avg | CPU |  | Best | Avg | CPU |
| pr01 | 190.02 | 190.02 | 3.06 |  | 190.02 | 190.02 | 5.79 |  | 190.02 | 190.02 | 8.47 |  | 190.02 | 190.02 | 11.20 |
| pr02 | 301.34 | 302.73 | 7.30 |  | 301.16 | 302.50 | 13.85 |  | 301.16 | 302.50 | 20.15 |  | 301.16 | 302.42 | 26.23 |
| pr03 | 534.27 | 538.12 | 6.32 |  | 529.15 | 537.62 | 11.29 |  | 529.15 | 537.62 | 15.85 |  | 529.15 | 537.62 | 19.96 |
| pr04 | 582.39 | 586.59 | 10.70 |  | 576.81 | 581.87 | 14.52 |  | 576.81 | 581.87 | 18.63 |  | 576.81 | 580.78 | 22.99 |
| pr05 | 639.13 | 644.52 | 13.77 |  | 636.61 | 644.43 | 22.72 |  | 636.61 | 644.43 | 31.54 |  | 636.61 | 641.52 | 40.07 |
| pr06 | 806.24 | 814.51 | 16.25 |  | 796.72 | 811.24 | 27.30 |  | 796.72 | 811.24 | 38.06 |  | 796.72 | 808.24 | 48.73 |
| pr07 | 291.71 | 292.96 | 4.32 |  | 291.71 | 291.71 | 8.25 |  | 291.71 | 291.71 | 12.45 |  | 291.71 | 291.71 | 16.69 |
| pr08 | 494.73 | 502.37 | 5.92 |  | 491.74 | 501.86 | 10.40 |  | 491.74 | 501.86 | 17.24 |  | 491.74 | 501.86 | 24.59 |
| pr09 | 665.28 | 668.91 | 4.29 |  | 662.31 | 668.91 | 9.81 |  | 661.83 | 668.91 | 17.83 |  | 661.83 | 668.91 | 25.80 |
| pr10 | 871.48 | 881.73 | 15.45 |  | 866.44 | 880.42 | 23.57 |  | 866.47 | 880.42 | 30.57 |  | 864.27 | 879.72 | 37.37 |
| pr11 | 164.46 | 165.37 | 2.80 |  | 164.46 | 164.51 | 4.77 |  | 164.46 | 164.51 | 6.76 |  | 164.46 | 164.51 | 8.73 |
| pr12 | 296.83 | 298.75 | 9.38 |  | 295.27 | 297.99 | 17.03 |  | 295.27 | 297.99 | 25.14 |  | 295.27 | 297.99 | 32.91 |
| pr13 | 490.42 | 496.05 | 9.56 |  | 485.47 | 493.57 | 15.81 |  | 485.43 | 493.57 | 22.15 |  | 485.43 | 493.57 | 28.60 |
| pr14 | 541.27 | 545.53 | 14.21 |  | 536.12 | 544.92 | 23.87 |  | 536.12 | 544.92 | 33.44 |  | 535.73 | 542.27 | 43.09 |
| pr15 | 589.47 | 592.72 | 22.65 |  | 584.71 | 592.16 | 36.38 |  | 584.71 | 592.16 | 50.36 |  | 582.46 | 590.74 | 64.02 |
| pr16 | 745.27 | 749.56 | 25.53 |  | 738.14 | 749.56 | 40.92 |  | 738.14 | 749.56 | 56.52 |  | 738.14 | 749.56 | 73.73 |
| pr17 | 248.21 | 252.38 | 1.35 |  | 248.21 | 251.98 | 1.61 |  | 248.21 | 251.96 | 1.89 |  | 248.21 | 251.40 | 2.46 |
| pr18 | 464.74 | 470.28 | 8.37 |  | 463.52 | 466.63 | 12.87 |  | 463.52 | 464.72 | 18.48 |  | 463.52 | 464.72 | 23.59 |
| pr19 | 605.83 | 612.97 | 11.22 |  | 604.52 | 611.34 | 12.04 |  | 604.54 | 611.34 | 13.15 |  | 600.27 | 609.58 | 17.01 |
| pr20 | 797.67 | 808.35 | 21.26 |  | 796.12 | 805.72 | 32.53 |  | 795.62 | 804.57 | 43.35 |  | 795.62 | 803.26 | 55.16 |
| **Average** | 516.04 | 520.72 | 10.69 |  | 512.96 | 519.45 | 17.27 |  | 512.91 | 519.29 | 24.10 |  | 512.46 | 518.52 | 31.15 |

In Table 11-Table 13, we present the detailed comparison between the LD-DA and each one of the compared methods. Each table consists of the LD-DA and the compared method performances. Then it is followed by the gap of best and average objective in percentage when the compared method performance is considered as the baseline. The last column of each table shows the ratio of compared methods computational time over the LD-DA CPU time.

Table 11- Detailed comparison of the LD-DA and ELS [36].

| instance | LD-DA (50 k iterations) | | |  | ELS | | |  | Gap | | |
| --- | --- | --- | --- | --- | --- | --- | --- | --- | --- | --- | --- |
|  | Best | Avg | CPU |  | Best | Avg | CPU |  | Best (%) | Avg (%) | CPU (ratio) |
| pr01 | 190.02 | 190.02 | 5.79 |  | 190.02 | 190.02 | 15.00 |  | 0.00 | 0.00 | 2.59 |
| pr02 | 301.16 | 302.50 | 13.85 |  | 301.34 | 301.34 | 75.00 |  | -0.06 | 0.38 | 5.42 |
| pr03 | 529.15 | 537.62 | 11.29 |  | 532.43 | 533.86 | 138.00 |  | -0.62 | 0.70 | 12.22 |
| pr04 | 576.81 | 581.87 | 14.52 |  | 570.54 | 574.47 | 442.20 |  | 1.10 | 1.29 | 30.45 |
| pr05 | 636.61 | 644.43 | 22.72 |  | 630.82 | 637.59 | 724.20 |  | 0.92 | 1.07 | 31.88 |
| pr06 | 796.72 | 811.24 | 27.30 |  | 792.80 | 796.10 | 1315.20 |  | 0.49 | 1.90 | 48.18 |
| pr07 | 291.71 | 291.71 | 8.25 |  | 291.71 | 292.96 | 28.20 |  | 0.00 | -0.43 | 3.42 |
| pr08 | 491.74 | 501.86 | 10.40 |  | 491.60 | 493.16 | 160.80 |  | 0.03 | 1.76 | 15.46 |
| pr09 | 662.31 | 668.91 | 9.81 |  | 672.86 | 681.35 | 671.00 |  | -1.57 | -1.83 | 68.40 |
| pr10 | 866.44 | 880.42 | 23.57 |  | 857.36 | 860.68 | 1279.80 |  | 1.06 | 2.29 | 54.30 |
| pr11 | 164.46 | 164.51 | 4.77 |  | 164.46 | 164.46 | 16.80 |  | 0.00 | 0.03 | 3.52 |
| pr12 | 295.27 | 297.99 | 17.03 |  | 295.66 | 295.72 | 82.20 |  | -0.13 | 0.77 | 4.83 |
| pr13 | 485.47 | 493.57 | 15.81 |  | 489.00 | 490.70 | 222.20 |  | -0.72 | 0.58 | 14.05 |
| pr14 | 536.12 | 544.92 | 23.87 |  | 531.08 | 531.98 | 612.00 |  | 0.95 | 2.43 | 25.64 |
| pr15 | 584.71 | 592.16 | 36.38 |  | 578.44 | 580.23 | 1195.80 |  | 1.08 | 2.06 | 32.87 |
| pr16 | 738.14 | 749.56 | 40.92 |  | 731.25 | 736.59 | 1939.20 |  | 0.94 | 1.76 | 47.39 |
| pr17 | 248.21 | 251.98 | 1.61 |  | 248.21 | 248.21 | 34.80 |  | 0.00 | 1.52 | 21.61 |
| pr18 | 463.52 | 466.63 | 12.87 |  | 461.21 | 462.40 | 259.20 |  | 0.50 | 0.91 | 20.14 |
| pr19 | 604.52 | 611.34 | 12.04 |  | 595.39 | 597.53 | 745.80 |  | 1.53 | 2.31 | 61.94 |
| pr20 | 796.12 | 805.72 | 32.53 |  | 796.60 | 803.99 | 1887.00 |  | -0.06 | 0.22 | 58.01 |
| **Average** | 512.96 | 519.45 | 17.27 |  | 511.14 | 513.67 | 592.22 |  | 0.27 | 0.99 | 28.12 |

Table 12- Detailed comparison of the LD-DA and Pure ALNS [29].

| instance | LD-DA (50 k iterations) | | |  | Pure ALNS | | |  | Gap | | |
| --- | --- | --- | --- | --- | --- | --- | --- | --- | --- | --- | --- |
|  | Best | Avg | CPU |  | Best | Avg | CPU |  | Best (%) | Avg (%) | CPU (ratio) |
| pr01 | 190.02 | 190.02 | 5.79 |  | 190.02 | 190.02 | 8.20 |  | 0.00 | 0.00 | 1.42 |
| pr02 | 301.16 | 302.50 | 13.85 |  | 301.34 | 301.34 | 16.70 |  | -0.06 | 0.38 | 1.21 |
| pr03 | 529.15 | 537.62 | 11.29 |  | 532.00 | 532.37 | 28.80 |  | -0.54 | 0.99 | 2.55 |
| pr04 | 576.81 | 581.87 | 14.52 |  | 571.41 | 574.34 | 48.10 |  | 0.95 | 1.31 | 3.31 |
| pr05 | 636.61 | 644.43 | 22.72 |  | 632.44 | 635.26 | 69.80 |  | 0.66 | 1.44 | 3.07 |
| pr06 | 796.72 | 811.24 | 27.30 |  | 792.97 | 795.25 | 95.30 |  | 0.47 | 2.01 | 3.49 |
| pr07 | 291.71 | 291.71 | 8.25 |  | 291.71 | 291.71 | 10.20 |  | 0.00 | 0.00 | 1.24 |
| pr08 | 491.74 | 501.86 | 10.40 |  | 491.97 | 493.36 | 30.10 |  | -0.05 | 1.72 | 2.89 |
| pr09 | 662.31 | 668.91 | 9.81 |  | 660.55 | 665.63 | 55.30 |  | 0.27 | 0.49 | 5.64 |
| pr10 | 866.44 | 880.42 | 23.57 |  | 855.73 | 862.35 | 96.50 |  | 1.25 | 2.10 | 4.09 |
| pr11 | 164.46 | 164.51 | 4.77 |  | 164.46 | 164.46 | 9.40 |  | 0.00 | 0.03 | 1.97 |
| pr12 | 295.27 | 297.99 | 17.03 |  | 295.96 | 296.52 | 18.80 |  | -0.23 | 0.50 | 1.10 |
| pr13 | 485.47 | 493.57 | 15.81 |  | 485.82 | 488.68 | 33.80 |  | -0.07 | 1.00 | 2.14 |
| pr14 | 536.12 | 544.92 | 23.87 |  | 532.16 | 534.85 | 57.00 |  | 0.74 | 1.88 | 2.39 |
| pr15 | 584.71 | 592.16 | 36.38 |  | 582.07 | 584.07 | 89.00 |  | 0.45 | 1.39 | 2.45 |
| pr16 | 738.14 | 749.56 | 40.92 |  | 739.67 | 742.80 | 117.60 |  | -0.21 | 0.91 | 2.87 |
| pr17 | 248.21 | 251.98 | 1.61 |  | 248.21 | 248.21 | 11.40 |  | 0.00 | 1.52 | 7.08 |
| pr18 | 463.52 | 466.63 | 12.87 |  | 461.12 | 463.51 | 35.00 |  | 0.52 | 0.67 | 2.72 |
| pr19 | 604.52 | 611.34 | 12.04 |  | 596.91 | 599.33 | 69.60 |  | 1.27 | 2.00 | 5.78 |
| pr20 | 796.12 | 805.72 | 32.53 |  | 785.70 | 793.10 | 114.70 |  | 1.33 | 1.59 | 3.53 |
| **Average** | 512.96 | 519.45 | 17.27 |  | 510.61 | 512.86 | 50.77 |  | 0.34 | 1.10 | 3.05 |

Table 13- Detailed comparison of the LD-DA and Best configuration ALNS [29].

| instance | LD-DA (50 k iterations) | | |  | Best configuration ALNS | | |  | Gap | | |
| --- | --- | --- | --- | --- | --- | --- | --- | --- | --- | --- | --- |
|  | Best | Avg | CPU |  | Best | Avg | CPU |  | Best (%) | Avg (%) | CPU (ratio) |
| pr01 | 190.02 | 190.02 | 5.79 |  | 190.02 | 190.02 | 14.50 |  | 0.00 | 0.00 | 2.50 |
| pr02 | 301.16 | 302.50 | 13.85 |  | 301.34 | 301.34 | 38.40 |  | -0.06 | 0.38 | 2.77 |
| pr03 | 529.15 | 537.62 | 11.29 |  | 532.00 | 532.00 | 55.20 |  | -0.54 | 1.06 | 4.89 |
| pr04 | 576.81 | 581.87 | 14.52 |  | 570.29 | 570.86 | 142.20 |  | 1.14 | 1.93 | 9.79 |
| pr05 | 636.61 | 644.43 | 22.72 |  | 629.14 | 632.67 | 296.22 |  | 1.19 | 1.86 | 13.04 |
| pr06 | 796.72 | 811.24 | 27.30 |  | 788.86 | 790.25 | 370.00 |  | 1.00 | 2.66 | 13.55 |
| pr07 | 291.71 | 291.71 | 8.25 |  | 291.71 | 291.71 | 19.00 |  | 0.00 | 0.00 | 2.30 |
| pr08 | 491.74 | 501.86 | 10.40 |  | 489.89 | 491.44 | 70.70 |  | 0.38 | 2.12 | 6.80 |
| pr09 | 662.31 | 668.91 | 9.81 |  | 658.90 | 661.37 | 151.20 |  | 0.52 | 1.14 | 15.41 |
| pr10 | 866.44 | 880.42 | 23.57 |  | 854.60 | 858.65 | 369.10 |  | 1.39 | 2.54 | 15.66 |
| pr11 | 164.46 | 164.51 | 4.77 |  | 164.46 | 164.46 | 16.50 |  | 0.00 | 0.03 | 3.46 |
| pr12 | 295.27 | 297.99 | 17.03 |  | 295.66 | 296.18 | 44.50 |  | -0.13 | 0.61 | 2.61 |
| pr13 | 485.47 | 493.57 | 15.81 |  | 484.83 | 485.15 | 94.90 |  | 0.13 | 1.74 | 6.00 |
| pr14 | 536.12 | 544.92 | 23.87 |  | 530.88 | 531.94 | 220.00 |  | 0.99 | 2.44 | 9.22 |
| pr15 | 584.71 | 592.16 | 36.38 |  | 576.88 | 577.27 | 469.60 |  | 1.36 | 2.58 | 12.91 |
| pr16 | 738.14 | 749.56 | 40.92 |  | 737.09 | 740.12 | 687.90 |  | 0.14 | 1.28 | 16.81 |
| pr17 | 248.21 | 251.98 | 1.61 |  | 248.21 | 248.21 | 22.10 |  | 0.00 | 1.52 | 13.73 |
| pr18 | 463.52 | 466.63 | 12.87 |  | 461.48 | 461.64 | 99.70 |  | 0.44 | 1.08 | 7.75 |
| pr19 | 604.52 | 611.34 | 12.04 |  | 594.14 | 596.02 | 343.50 |  | 1.75 | 2.57 | 28.53 |
| pr20 | 796.12 | 805.72 | 32.53 |  | 784.57 | 790.01 | 472.20 |  | 1.47 | 1.99 | 14.52 |
| **Average** | 512.96 | 519.45 | 17.27 |  | 509.25 | 510.57 | 199.87 |  | 0.56 | 1.48 | 10.11 |
